# Gene expression profiling of gliomas: merging genomic and histopathological classification for personalised therapy

**DOI:** 10.1038/sj.bjc.6606031

**Published:** 2010-11-30

**Authors:** M Vitucci, D N Hayes, C R Miller

**Affiliations:** 1 Curriculum in Genetics and Molecular Biology, University of North Carolina School of Medicine, Chapel Hill, NC, USA; 2 Lineberger Comprehensive Cancer Center, University of North Carolina School of Medicine, Chapel Hill, NC, USA; 3 Division of Hematology-Oncology, Department of Internal Medicine, University of North Carolina School of Medicine, Chapel Hill, NC, USA; 4 Division of Neuropathology, Department of Pathology & Laboratory Medicine, University of North Carolina School of Medicine, Chapel Hill, NC, USA; 5 Neurosciences Center, University of North Carolina School of Medicine, Chapel Hill, NC, USA

**Keywords:** glioma, glioblastoma, gene expression profiling, microarrays, personalised therapy, prognosis

## Abstract

The development of DNA microarray technologies over the past decade has revolutionised translational cancer research. These technologies were originally hailed as more objective, comprehensive replacements for traditional histopathological cancer classification systems, based on microscopic morphology. Although DNA microarray-based gene expression profiling (GEP) remains unlikely in the near term to completely replace morphological classification of primary brain tumours, specifically the diffuse gliomas, GEP has confirmed that significant molecular heterogeneity exists within the various morphologically defined gliomas, particularly glioblastoma (GBM). Herein, we provide a 10-year progress report on human glioma GEP, with focus on development of clinical diagnostic tests to identify molecular subtypes, uniquely responsive to adjuvant therapies. Such progress may lead to a more precise classification system that accurately reflects the cellular, genetic, and molecular basis of gliomagenesis, a prerequisite for identifying subsets uniquely responsive to specific adjuvant therapies, and ultimately in achieving individualised clinical care of glioma patients.

Morphological evaluation of cancers by light microscopy has been the foundation for diagnosis, prognostication, and therapeutic stratification for well over a century. However, patients with morphologically identical tumours can have significantly different clinical outcomes. To address the pressing medical need for more accurate predictions, a variety of transformative technologies have been developed over the last four decades – electron microscopy, molecular biology, immunohistochemistry, and quantitative RT–PCR – to refine traditional cancer classification or as outright replacements. The newest such technology, DNA microarrays, was introduced in 1995, and its potential clinical utility in oncology was quickly recognised. In fact, the Director of the US National Cancer Institute issued a challenge to the scientific community in 1999 (NCI, 1999) to ‘harness the power of comprehensive molecular analysis technologies to make the classification of tumours vastly more informative. This challenge is intended to lay the groundwork for *changing the basis* (emphasis added) of tumour classification from morphological to molecular characteristics.’

The response from the cancer research community has been intense: nearly 14 000 publications have utilised DNA microarrays for genome-wide gene expression profiling (GEP) in all aspects of cancer research, from basic to translational to clinical. GEP has unequivocally established that significant molecular heterogeneity exists within morphologically defined cancers and that potentially clinically relevant molecular subtypes can be identified. However, to date, only two molecular diagnostic tests, developed using DNA microarrays, have either been approved by the US Food and Drug Administration (MammaPrint) or incorporated into practise guidelines (Oncotype Dx) for clinical use in breast cancer (Weigelt *et al*, 2009).

This discordance between scientific productivity and clinical implementation over the course of a decade is not unexpected, given the stringent sample requirements, pace of technology development, data volume and complexity, continually evolving data analysis techniques, lack of defined best practices for analysis, and levels of evidence required for clinical use. A number of excellent review articles have discussed these and other impediments in implementing GEP clinically (Dupuy and Simon, 2007; Weigelt *et al*, 2009; Subramanian and Simon, 2010). Herein, we review a decade of DNA microarray-based GEP on the most common and biologically aggressive group of primary brain tumours, the diffuse gliomas (hereafter referred as gliomas). The discussion will re-visit morphological classification and address the potential role of GEP in identifying clinically relevant molecular subtypes of gliomas. We will then primarily focus on studies that have examined the prognostic impact of multi-gene signatures for the most deadly glioma, glioblastoma (GBM).

## Morphological classification of gliomas

Bailey and Cushing established the first diagnostic classification system for primary brain tumours in 1926, based on their understanding of the histogenetic basis of brain development and the morphological resemblance of primary brain tumours to their presumed developmental counterparts by light microscopy. This system has been refined periodically, culminating in the current World Health Organization (WHO) scheme (Louis *et al*, 2007). Seven gliomas are currently recognised as distinct clinicopathological entities, each characterised by cytological and immunohistochemical evidence of differentiation along astrocytic, oligodendroglial, or both glial lineages (Table 1). Further refinement into distinct prognostic groups is dictated by histological grading (II–IV), based on morphological features associated with more aggressive biology, including mitoses, microvascular proliferation, and necrosis (Miller and Perry, 2007). Molecular and genetic features constitute an additional level of detail utilised not only to diagnostically differentiate among these entities but also increasingly to predict clinical outcomes and response to adjuvant therapies.

**Figure 1:**
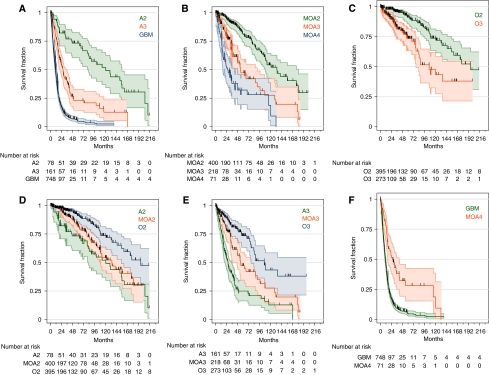
Overall survival of patients with newly diagnosed gliomas, grouped on the basis of the two main components of the WHO classification system: differentiation (cytology) – astrocytic (**A**), mixed oligoastrocytic (**B**), or oligodendroglial (**C**); and histological grade – WHO grade II (**D**), III (**E**), or IV (**F**). Clinicopathological parameters, statistics, and abbreviations are listed in Table 1.

The prognostic power of the current WHO glioma classification has facilitated its widespread adoption for clinical patient management. However, it has long been recognised that individual patients within each diagnostic category can have vastly different outcomes that are not otherwise accounted for by established prognostic factors, including age, Karnofsky performance status (KPS), and therapy. This prognostic variability can be visualised using the 95% confidence intervals of Kaplan–Meier survival curves (Figure 1). The extent to which prognostic factors account for outcome variability in multivariate Cox proportional hazards models can be quantified with metrics such as Harrell's C statistics (Table 1) (Miller *et al*, 2006). Using these two measurements, prognostic variability is least pronounced in astrocytic gliomas (Figure 1A), particularly GBM, and is substantially higher in mixed (Figure 1B) and pure oligodendroglial (Figure 1C) gliomas. Prognostic variability is most pronounced among the lower-grade gliomas (Figures 1D and E). For these gliomas in particular, accurate classification and prognostication have become increasingly dependent on molecular assays. The most notable test detects co-deletion of chromosomal arms 1p and 19q, a genetic signature and favourable prognostic factor, strongly associated with oligodendroglial differentiation (Miller *et al*, 2006). However, even with ancillary molecular testing, classification of a subset of morphologically ambiguous grade II and III gliomas remains challenging, even among experienced neuropathologists (Miller *et al*, 2006; Miller and Perry, 2007). Clearly, more objective, molecular methods for diagnostic discrimination among gliomas are needed.

The clinicopathological variables central to the WHO 2007 classification – patient age at diagnosis, differentiation (cytology), histological grade, and 1p19q co-deletion status – account for 70–80% of the prognostic variability among each of the three major types of gliomas, based on the C index (Table 1). Inclusion of additional clinical factors (e.g., KPS, therapy) not otherwise available in this retrospective data set would likely account for even more of the prognostic variability. Despite the inability to accurately predict outcomes for individual patients, this example clearly illustrates that existing clinicopathological factors account for the vast majority of prognostic variability in gliomas. It is in this context – the ability to provide prognostic information independent of established factors – that the clinical utility of GEP must be defined (Dupuy and Simon, 2007). The key for clinical implementation of GEP will therefore be to quantify the remaining 20–30% of prognostic variability by one of two means: (1) utilising GEP as a diagnostic adjunct to more accurately classify morphologically ambiguous gliomas, or (2) to identify prognostically distinct molecular subtypes within otherwise morphologically homogeneous gliomas.

## Molecular classification of gliomas

The earliest GEP studies utilised class comparison to identify differentially expressed genes among morphologically defined gliomas. Such genes were found in low-grade *vs* high-grade astrocytomas (Rickman *et al*, 2001), high-grade oligodendrogliomas *vs* GBM (Nutt *et al*, 2003; Shirahata *et al*, 2007), primary *vs* secondary GBM (Godard *et al*, 2003; Shai *et al*, 2003; Tso *et al*, 2006), adult *vs* paediatric GBM (Faury *et al*, 2007), or a variety of morphologically defined glioma subtypes (Godard *et al*, 2003; Shai *et al*, 2003; van den Boom *et al*, 2003). Using primarily hierarchical clustering on differentially expressed genes, transcriptomal profiles of individual tumours were shown to be most similar to those from the same diagnostic category, that is, gliomas of similar differentiation and grade. These studies confirmed that morphological differences among gliomas are reflected at the mRNA transcript level and that differentially expressed genes could be utilised to distinguish among morphologically defined subtypes. However, discordance between morphological diagnosis and GEP-defined molecular subtype was frequent, likely due in part to inclusion of difficult to classify morphologically ambiguous gliomas.

Nutt, Louis, and colleagues provided a glimpse of the potential clinical utility of GEP as an ancillary diagnostic test for more accurate glioma classification (Nutt *et al*, 2003). These investigators identified genes significantly correlated with either morphologically classical GBM or anaplastic oligodendroglioma in a training set of 21 tumours, and built a class prediction model that showed 86% accuracy in assigning 29 diagnostically challenging GBM and anaplastic oligodendrogliomas to their respective diagnostic categories. More importantly, a statistically significant difference in overall survival for the GEP but not the morphologically defined groups was found, suggesting that GEP may provide more accurate classification and prognostication, particularly for morphologically ambiguous gliomas. These findings were confirmed by Shirahata *et al* (2007), who identified 168 differentially expressed genes from PCR array data on 32 GBM and anaplastic oligodendrogliomas, and used a weighted voting algorithm to develop a 67-gene diagnostic assay with 96.6% accuracy in distinguishing between these two prognostically distinct high-grade gliomas from the published Nutt data set (Nutt *et al*, 2003).

Li, Fine, and colleagues provided the first report of a comprehensive, molecular classification of all gliomas (Li *et al*, 2009). These authors utilised two unsupervised machine-learning methods on a large training set (*N*=159) of WHO grade II–IV gliomas taken from all three histological categories. Guided only by molecular data, without influence of prior morphological diagnosis, they identified six hierarchically nested subtypes, divided into two main categories (O and G). The first category contained two subgroups (OA and OB) and the second had four nested subgroups (GA1, GA2, GB1, and GB2). These data confirmed that morphological differences among gliomas are reflected at the mRNA transcript level. Survival analyses showed that the O and G main groups and the OA and OB subgroups of O-type tumours, but not the four G subgroups, were prognostically distinct. Importantly, the prognostic impact of the two main subgroups was confirmed in an independent data set from The Cancer Genome Atlas (TCGA), consisting entirely of GBM (Verhaak *et al*, 2010), whereas that of the six subgroups was confirmed in the REMBRANDT (Madhavan *et al*, 2009) and Phillips *et al* (2006) data sets consisting of all seven gliomas. However, the concordance between GEP-defined subtypes and histopathological diagnoses was not assessed and multivariate survival analyses with known prognostic factors were not conducted.

In retrospect, the aforementioned studies utilised small (*N*<100 per diagnostic category), ostensibly convenience cohorts of previously banked, frozen gliomas. As such, individual studies were statistically underpowered to assess the diagnostic discriminatory power of GEP *vis-à-vis* morphological classification. Moreover, the relatively small sample sizes and lack of data on known prognostic covariates precluded comprehensive multivariable analyses. Particularly for the earlier studies, the prognostic impact of GEP signatures could not be validated in large, external data sets (Subramanian and Simon, 2010). Fortunately, most data have been deposited in publically available online repositories, including the Gene Expression Omnibus and REMBRANDT (Madhavan *et al*, 2009). These data have already been instrumental in both novel hypothesis-driven, mechanistic studies (Brennan *et al*, 2009) and subsequent GEP studies described below. Only through collection of GEP data on a sufficient number of all seven morphologically defined gliomas will it be possible to assess whether GEP will be diagnostically robust enough to replace morphology as the basis for glioma classification.

## GEP identifies prognostically distinct molecular subtypes of gliomas

A number of GEP studies have identified prognostically distinct molecular subtypes of gliomas. In 2004, Freije, Nelson, and colleagues analysed 74 gliomas from four histological types and identified 595 differentially expressed genes that correlated with overall survival (Freije *et al*, 2004). Hierarchical clustering showed four molecular subtypes (labelled HC1A, HC1B, HC2A, and HC2B) that segregated into two distinct (*P*=0.00011) survival clusters (SCs): SC1 (93% HC1A/B and 62% non-GBM) and SC2 (76% HC2A/B and 89% GBM) with 4.8 and 0.6 years (y) median overall survival, respectively. Prognostic significance was confirmed in the independent Nutt data set (Nutt *et al*, 2003), and multivariate analysis showed that survival cluster was independent of patient age and histological grade. Functional annotation of the gene lists showed that HC1A subtype tumours were enriched for genes involved in neurogenesis (Kriegstein and Alvarez-Buylla, 2009), suggesting a more differentiated phenotype. In contrast, the poor survival subtypes were enriched for proliferation (HC2A) and extracellular matrix/invasion-related (HC2B) genes. A similar list of survival-related genes implicated in neurogenesis was identified by Liang *et al* (2005), who also showed that GBM could be divided into two prognostically distinct molecular subtypes (median overall survival 2.1 *vs* 0.3 y).

In 2006, Phillips, Aldape, and colleagues analysed 76 high-grade astrocytomas and identified 108 differentially expressed genes significantly associated with overall survival (Phillips *et al*, 2006). Hierarchical and k-means clustering with those genes showed three distinct subtypes termed as proneural, proliferative, and mesenchymal, based on functional annotation of representative genes. Like Frieje HC1A, the proneural subtype was defined by genes implicated in neurogenesis, composed predominantly (69%) of non-GBM, and associated with significantly more favourable median overall survival (3.6 *vs* ⩽1.3 y), independent of histological grade. In contrast, the proliferative and mesenchymal gene signatures were enriched for proliferation- and extracellular matrix/invasion-related genes, similar to the Frieje HC2A and HCA2B subtypes, respectively. Prognostic significance of molecular subtype was validated in an independent cohort of 184 gliomas of various histological types. Taken together, these results suggest that (1) the molecular subtype of a majority of WHO grade II-III gliomas is HC1A/proneural, and (2) HC1A/proneural GBM may be more prognostically favourable.

Using published data sets and new GEP data on 86 GBM, a subsequent meta-analysis by Lee *et al* (2008) utilised 377 differentially expressed genes that divided GBM into four distinct subtypes on hierarchical clustering: HC1A/proneural, HC2A/proliferative, HC2B/mesenchymal, and a fourth with hybrid HC2A/HC2B features termed ProMes. Survival analysis confirmed the more favourable prognosis of HC1A/proneural GBM *vs* the remaining three molecular subtypes (median 1.4 *vs* 0.9 y). With this larger data set of 267 GBM, the authors also confirmed an association first identified by Phillips *et al* (2006), namely that the mean age at diagnosis of proneural GBM patients was significantly younger (51 *vs* 55 y, *P*=0.02). Moreover, in multivariable analyses, only molecular subtype, but not age, was significantly associated with overall survival. These data suggest a molecular basis for the known association of younger age with improved overall survival in GBM patients.

However, it is of critical note that none of these prognostic studies distinguished among recognised morphological variants of GBM. As shown in Table 1, GBM with oligodendroglial features occur in younger patients and have a significantly prolonged overall survival compared with their GBM counterparts (*P*<0.0001). Similarly, another morphological variant of GBM, small-cell GBM (Miller and Perry, 2007), characterised by frequent gains of chromosome 7 (*EGFR*) and loss of chromosome 10q (*PTEN*), is morphologically similar to the prognostically more favourable anaplastic oligodendroglioma, but lacks 1p19q co-deletion. The recent recognition of these morphological patterns of GBM (Louis *et al*, 2007; Miller and Perry, 2007), prognostically distinct from anaplastic oligoastrocytoma and anaplastic oligodendroglioma, respectively, raises the possibility that earlier studies were ‘contaminated’ with tumours known to have different prognoses. In addition, at least two significant design flaws were common in these studies (Dupuy and Simon, 2007; Subramanian and Simon, 2010): (1) subtype-specific signature genes were identified using heterogeneous training sets composed of various histological subtypes (e.g., anaplastic astrocytoma and GBM) with known differences in overall survival (Table 1) and (2) signature genes were defined on the basis of their association with outcome in training sets, and their prognostic significance was re-analyzed in independent test sets, raising the possibility that the correlation between GEP-defined subtypes and overall survival were a consequence of prior selection for outcome-related genes (Dupuy and Simon, 2007). To avoid the first problem, future studies should ideally define prognostic signatures in morphologically- and hence prognostically homogeneous cohorts of gliomas. Moreover, consensus diagnosis among multiple, experienced neuropathologists and/or utilisation of ancillary molecular testing such as 1p19q status for accurate assignment of morphologically ambiguous cases into established diagnostic categories will be important quality control measures.

The second problem is likely mitigated by two recently published studies that have identified the HC1A/proneural subset of GBM using gene signatures defined completely by unsupervised methods. In the largest single-institution study conducted to date, Gravendeel *et al* (2009) defined molecular subtypes for 276 gliomas of all histological types. Using 5000 genes with highly variable expression, these authors identified six molecular subtypes with distinct prognoses. Glioblastoma largely (73–86%) fell into three clusters (18, 22, and 23) and these tumours showed inferior prognosis relative to GBM in other clusters (9, 16, and 17) (median overall survival 0.7 *vs* 2.1 y). Cluster 9 consisted primarily (86%) of oligodendroglial neoplasms and the vast majority (82%) appropriately harboured combined 1p19q loss-of-heterozygosity. Notably, the prognostically superior cluster 17 (median overall survival 3.3 and 2.1 y for all C17 gliomas and GBM, respectively) significantly (97%) overlapped with the Phillips proneural subtype, suggesting that detection of a subgroup of GBM with improved prognosis and transcriptional profiles similar to lower-grade gliomas was not a consequence of prior selection of outcome-related genes (Phillips *et al*, 2006). Notably, cluster 22 was enriched (38%) for secondary GBM, tumours that progress from lower-grade precursors, arise in younger patients (Miller and Perry, 2007), and feature *IHD1* mutations (Yan *et al*, 2009), but lack *EGFR* amplification (Louis *et al*, 2007). These findings confirm those from a previous study that demonstrated distinct molecular profiles in primary *vs* secondary GBM (Tso *et al*, 2006). Clusters 18 and 23 contained predominantly GBM (78 and 86%, respectively) and showed significant overlap with Phillips proliferative (52%) and mesenchymal (93%) subtypes (Phillips *et al*, 2006). On analysis of data (Murat *et al*, 2008) from the definitive phase III clinical trial that established concomitant chemoradiotherapy and adjuvant temozolomide as the standard-of-care for newly diagnosed GBM patients (Stupp *et al*, 2005), these clusters were found to selectively benefit from combined chemoradiation *vs* radiation alone. Importantly, multivariate analysis included most known prognostic factors, including age, gender, histological type, grade, KPS, surgery, chemotherapy, *EGFR* amplification, 1p19q status, and *IDH1* mutation (Yan *et al*, 2009). Only molecular subtype, KPS, and gender were significant, independent prognostic factors in this data set (*P*⩽0.02), suggesting that molecular subtyping may be more prognostically accurate than morphological classification. Moreover, these authors validated the prognostic significance of their signatures in four independent data sets (Phillips *et al*, 2006; TCGA, 2008; Li *et al*, 2009; Madhavan *et al*, 2009).

The TCGA, established by the US National Cancer Institute and National Human Genome Research Institute in December 2005, with the mission of understanding ‘the molecular basis of cancer through the application of genome analysis technologies,’ selected GBM as its first cancer type for study, based on its uniformly poor prognosis and limited treatment options. As part of this multi-institutional project, we analysed 200 GBM on three different GEP platforms (Verhaak *et al*, 2010). Unsupervised hierarchical cluster analysis defined four subtypes, termed proneural, neural, classical, and mesenchymal, based on functional gene annotation and prior convention (Phillips *et al*, 2006). Significant overlap in molecular subtypes was found for TCGA mesenchymal/Phillips mesenchymal/Freije HC2B and TCGA proneural/Phillips proneural/Freije HC1A (Freije *et al*, 2004; Phillips *et al*, 2006). Unlike previous studies, the TCGA proneural subtype was not associated with improved prognosis in the TCGA data set consisting solely of GBM, but was in the validation of the data sets (Phillips *et al*, 2006; Madhavan *et al*, 2009) containing lower-grade gliomas. Conversely, re-analysis of the TCGA GBM data with Phillips molecular subtype designations confirmed a slightly more favourable prognosis of the Phillips proneural GBM (median overall survival 1.2 y) relative to Phillips mesenchymal/proliferative GBM subtypes (1.0 and 0.6 y, respectively, *P*=0.03). These findings suggest that subtyping based on prognosis-defined, but not ‘intrinsic’, unsupervised gene signatures may identify a subset of GBM with more favourable prognosis. However, similar to previous findings (Gravendeel *et al*, 2009), the TCGA classical and mesenchymal subtypes showed significantly improved overall survival after conventional chemoradiation or ⩾4 cycles of cytotoxic chemotherapy (*P*=0.02), suggesting that these subtypes may be particularly sensitive to DNA-damaging agents. These hypotheses will be tested further in two ongoing phase III clinical trials conducted by the Radiation Therapy Oncology Group (RTOG), as discussed below.

Capitalising on the unprecedented level of molecular data available for these tumours (TCGA, 2008), we identified recurrent genomic aberrations in each molecular subtype. The classical subtype was characterised by frequent *EGFR* amplification and EGFRvIII mutations, *CDKN2A* deletion, and a lack of *TP53* mutations, whereas the mesenchymal subtype was characterised by *NF1*, *TP53*, and *PTEN* mutations. Consensus neuropathological review of a subset of TCGA cases has shown that the proneural, classical, and mesenchymal subtypes are enriched for GBM with oligodendroglial features, small-cell GBM, and gliosarcoma (a morphological variant of GBM with mesenchymal differentiation (Miller and Perry, 2007)), respectively (Cameron Brennan, personal communication). Moreover, pseudopalisading necrosis and to a lesser extent florid microvascular proliferation are frequent in mesenchymal GBM, but the proneural subtype typically lacks necrosis. These findings suggest that mesenchymal GBM may be uniquely susceptible to angiogenesis inhibitors, a hypothesis currently being tested in the RTOG 0825 trial discussed below. The proneural subtype, which like previous studies (Phillips *et al*, 2006; Lee *et al*, 2008) was found in younger patients, harboured frequent *PDGFRA* amplification and mutations in *IDH1*, *TP53*, and *PIK3CA*/*PIK3R1*, suggesting susceptibility to PDGFRA- and PI3K-targeted therapies. A recent proteomic analysis confirmed protein- and phosphorylation-level signalling abnormalities in the EGFR, PDGFR, and NF1 pathways in classical, proneural, and mesenchymal subtypes of GBM, respectively, further suggesting that these GBM subtypes may be uniquely susceptible to targeted agents (Brennan *et al*, 2009).

A recent TCGA effort utilised methylation profiling to identify a GBM CpG island methylator phenotype (G-CIMP) in a significant fraction (29%) of proneural GBM, particularly secondary, *IDH1* mutation-positive GBM that progressed from lower-grade tumours (Noushmehr *et al*, 2010). This implies that G-CIMP might be common in lower-grade gliomas, the majority of which cluster with the proneural molecular subtype of GBM (Phillips *et al*, 2006; Gravendeel *et al*, 2009). To further investigate this hypothesis, Noushmehr and colleagues analysed eight G-CIMP gene regions in seven hypermethylated loci in an independent cohort of 152 WHO grade II and III gliomas by a MethyLight real-time PCR assay and found 46% of astrocytomas and 93% of oligodendrogliomas to be G-CIMP-positive. Furthermore, G-CIMP-positive GBM patients were younger (median 36 *vs* 59 y, *P*<0.0001) and survived longer than G-CIMP-negative GBM of both proneural and non-proneural subtypes (median overall survival 2.9 *vs* 0.8 and 1.0 y, *P*=7E−7). Importantly, G-CIMP positivity was independent of age and histological grade on multivariable analysis. These findings suggest that G-CIMP defines a subset of proneural GBM and can be utilised to further refine expression-defined subtypes. The co-occurrence of G-CIMP/*IDH1* mutation positivity in the proneural, neurogenesis-related subtype further suggests that *IDH1* mutation and/or G-CIMP may confer neoplastic susceptibility to a common neuron/oligodendrocyte precursor cell of origin (Kriegstein and Alvarez-Buylla, 2009), a hypothesis supported by the comparative expression profiling data that showed enrichment of genes expressed in purified, cultured murine oligodendrocytes in proneural GBM (Verhaak *et al*, 2010).

## Clinical implementation of GEP for glioma classification

GEP-based diagnostic tests are currently being evaluated in prospective, randomised clinical trials in breast cancer (Weigelt *et al*, 2009). Similar progress in clinical neuro-oncology has recently been made. On the basis of a previous report (Phillips *et al*, 2006), Colman *et al* (2010) identified a consensus 38 gene signatures from four independent data sets and from this set chose nine genes (*AQP1*, *CHI3L1*, *EMP3*, *GPNMB*, *IGFBP2*, *LGALS3*, *OLIG2*, *PDPN*, and *RTN1*), based on their survival correlation and technical compatibility, for development of a quantitative, reverse transcription–polymerase chain reaction assay. On the basis of the logistical difficulties in obtaining fresh frozen tumours for DNA microarray-based assays, such an assay is absolutely critical for successful clinical implementation with formalin-fixed, paraffin-embedded (FFPE) GBM, which constitute the vast majority of clinical samples. The prognostic impact of this nine-gene profile was uniformly associated with both progression-free survival and overall survival, and independent of clinical (age and KPS) and molecular factors, including *MGMT* methylation status. This assay is currently being tested in two prospective, randomised, phase III clinical trials, conducted by the RTOG. RTOG0525 is investigating the use of dose-intensive adjuvant temozolomide *vs* standard of care (Stupp *et al*, 2005) in patients stratified on the basis of *MGMT* promoter methylation status. Prospectively banked FFPE tissue from this trial will be retrospectively analysed using the nine-gene predictor to confirm its prognostic significance relative to *MGMT* status in a uniformly treated patient population. RTOG0825 is investigating the benefit of adjuvant bevacizumab, a humanised, anti-angiogenesis monoclonal antibody, to standard of care and will prospectively randomise patients on the basis of both *MGMT* methylation status and the nine-gene assay. The study will address, as a secondary end point, the hypothesis that mesenchymal GBM will selectively benefit from the addition of bevacizumab to standard-of-care. Results from these important clinical trials are expected in 2011–2012. In summary, molecular subtyping now has the potential to become a readily implemented clinical test that may guide future treatment decisions, particularly in identifying those patients most likely to benefit from standard-of-care *vs* novel, molecularly targeted agents.

## Conclusion

As we have outlined above and summarised in Table 2, tremendous progress in DNA microarray-based GEP of gliomas has been made over the past decade. In the next decade, next-generation sequencing technologies such as RNA-seq (Wang *et al*, 2009) promises to accelerate the pace and depth of discovery, further strengthening GEP as a method for cancer classification by directly determining transcript identity, structure, and abundance at the single-base level. Although GEP has provided significant insights into the molecular heterogeneity of morphologically defined gliomas, its role in clinical neuro-oncology still remains to be established. Thus, 10 years after the challenge thrown by the director of the US National Cancer Institute, the need for a ‘vastly more informative classification system’ for gliomas still exists. In this review, we have argued that GEP and the established morphological classification system are complementary, not mutually exclusive. The most clinically appropriate uses of GEP will be as a diagnostic adjunct to more accurately classify morphologically ambiguous gliomas and the identification of molecular subtypes within otherwise morphologically homogeneous gliomas. There has been substantial progress in defining molecular subtypes of GBM. However, unlike commercially available genomic tests for breast cancer, molecular subtyping in GBM is unlikely to be utilised for risk stratification because of the limited prognostic variability of this tumour. Rather, as illustrated by the RTOG clinical trials, molecular subtyping in GBM shows promise in identifying subsets that may be uniquely responsive to specific adjuvant therapies. Thus, the recent merger of genomic and histopathological classification bodes well for the future of personalised medicine in neuro-oncology.

## Figures and Tables

**Table 1:** Prognostic utility of the WHO 2007 classification for diffuse gliomas

	**WHO grade**			**Multivariate analysis**			
	**II**	**III**	**IV**	**Prognostic factor**	**HR**	***P*-value**	**ΔC or overall C^a^**
*Astrocytomas*							
	DA, A2	AA, A3	GBM, A4				
*N*	78	161	748	Grade	1.9	<0.001	0.61
Median OS (y)	10.0	2.2	0.9	Age^b^	1.9	<0.001	0.08
95% CI	6.9–13.0	1.7–2.7	0.8–1.0	All (*N*=987)			0.69
Mean age	33	39	57				
Grading criteria		Mitoses	MVP with/without necrosis				
							
*Oligoastrocytomas*							
	OA, MOA2	AOA, MOA3	GBM-O, MOA4^c^				
*N*	400	218	71	1p19q codel	2.6	<0.001	0.54
Median OS (y)	11.1	3.9	2.2	Age^b^	2.1	<0.001	0.15
95% CI	9.0–15.0	2.8–4.6	1.3–3.4	Grade	2.2	0.007	0.10
Mean age	38	42	48	All (*N*=559)			0.79
Grading criteria		Mitoses with/without MVP	Necrosis				
							
*Oligodendrogliomas*							
	ODG, O2	AO, O3					
*N*	395	273		1p19q codel	2.1	0.020	0.54
Median OS (y)	16.4	8.8		Age^b^	2.4	<0.001	0.17
95% CI	12.9–21.1	6.5-ND		Grade	2.5	0.004	0.03
Mean age	40	44		All (*N*=539)			0.74
Grading criteria		Mitoses with/without MVP with/without necrosis					
							
*All diffuse gliomas*							
*N*		2344		1p19q codel	1.9	0.002	0.63
Median OS (y)		2.9		Age^b^	1.8	<0.001	0.13
95% CI		2.5–3.6		Cytology	1.7	<0.001	0.04
Mean age		46		Grade	2.0	<0.001	0.03
				All (*N*=1363)			0.83

Abbreviations: AA, A3=anaplastic astrocytomas; AO, O3=anaplastic oligodendroglioma; codel=co-deletion; CI=confidence interval; DA, A2=diffuse astrocytoma; HR=hazard ratio; GBM, A4=glioblastoma; GBM-O, MOA4=glioblastoma with oligodendroglial features; OA, MOA2=mixed oligoastrocytoma; AOA, MOA3=mixed anaplastic oligoastrocytoma; MVP=microvascular proliferation; ODG=olidodendroglioma; OS=overall survival; WHO=World HeALTH organization; y=years.

a Harrell's C statistic for the multivariable Cox proportional hazards model with all factors (C) or ΔC for each individual factor in the model Miller *et al* (2006).

b Age at diagnosis trichotomized as follows: ⩽40, 40–60, ⩾60 y Miller *et al* (2006).

c Note that GBM-O (MOA4) is not currently recognised as a distinct clinicopathological entity by the WHO; instead, it is considered a morphological pattern of GBM with a slightly more favourable prognosis Louis *et al* (2007).

Data from adult patients (⩾20 y) with newly diagnosed gliomas at Washington University School of Medicine (1977–2009 and Miller *et al* (2006)).

**Table 2:**
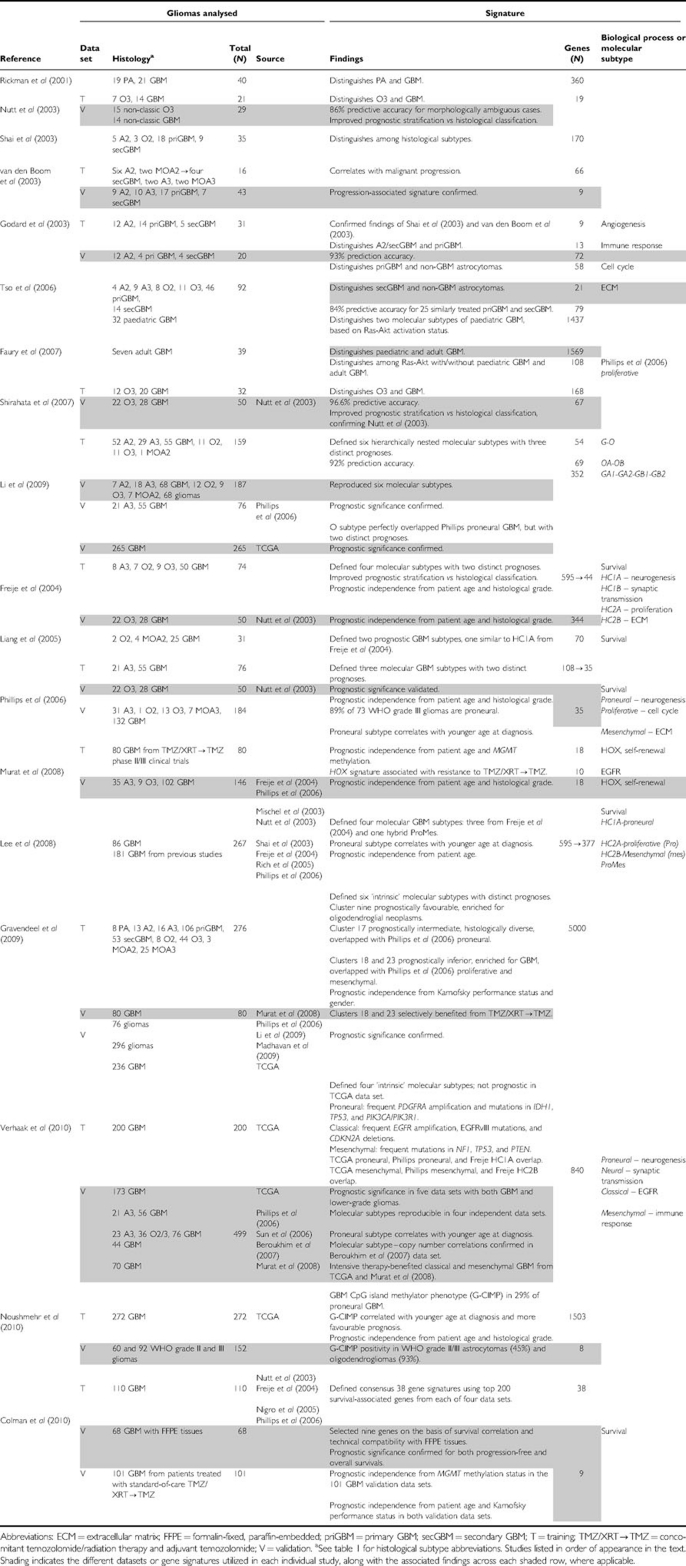
Summary of glioma microarray studies
